# Solid Lipid Nanoparticles by Coacervation from Natural Soaps: Preliminary Studies for Oral Delivery of an Insulin Analogue

**DOI:** 10.3390/pharmaceutics17101261

**Published:** 2025-09-26

**Authors:** Annalisa Bozza, Arianna Marengo, Federica Blua, Elisabetta Marini, Stefano Bagatella, Elena Ugazio, Elisabetta Muntoni, Luigi Battaglia

**Affiliations:** 1 Department of Drug Science and Technology, University of Turin, via Pietro Giuria 9, 10125 Turin, Italy; arianna.marengo@unito.it (A.M.); federica.blua@unito.it (F.B.); elisabetta.marini@unito.it (E.M.); elena.ugazio@unito.it (E.U.); elisabetta.muntoni@unito.it (E.M.); 2 Department of Veterinary Sciences, University of Turin, Largo Paolo Braccini 2, 10095 Grugliasco, Italy; stefano.bagatella@unito.it

**Keywords:** solid lipid nanoparticles, glargine insulin, coacervation, natural soaps, hydrophobic ion pairing

## Abstract

**Background/Objectives**: Oral insulin continues to constitute a challenge due to its low uptake by the gut wall and degradation by gastrointestinal proteolytic enzymes. Such concerns might be surmounted by means of nanoparticle delivery. **Methods**: In this study, glargine insulin has been loaded into solid lipid nanoparticles prepared via coacervation from Shea and Mango soaps, due to hydrophobic ion pairing. Subsequently, ex vivo tied-up-gut experiments were performed with fluorescently labeled peptide. Additionally, re-dispersible oral solid dosage forms (powders and tablets) were obtained from nanoparticle suspensions via freeze-drying and spray-drying. **Results**: Solid lipid nanoparticles are capable of enhancing peptide permeation into different gut sections. Furthermore, spray-drying permits the preparation, which can be scaled up, of a re-dispersible powder from the nanoparticle suspension. **Conclusions**: This engineered process is suitable for the formulation of solid oral dosage forms, such as granulates and tablets, and presents promising potential for oral insulin delivery, paving the way for the assessment of its pharmacological efficacy in further in vivo studies.

## 1. Introduction

According to a 2021 report by the International Diabetes Federation, there are approximately 537 million individuals with diabetes worldwide (nearly 10.5% of the world’s population), and almost half of them are still undiagnosed, especially in low- and middle-income countries. This poses several social and economic challenges for healthcare systems. Diabetes can be classified as type I, caused by a lack of insulin secretion by pancreatic β-cells, and type II, caused by reduced insulin tissue responsivity, which is strongly linked to obesity and a sedentary lifestyle, with there being a growing trend in occurrence over time in developed countries [1]. Insulin is a protein with 51 residues, consisting of two peptide chains (A with 21 and B with 30 amino acids) linked by two disulfide bonds and characterized by a molecular weight (M_W_) of nearly 5800 Dalton (Da). The administration of exogenous insulin is lifesaving for type I diabetes but still entails several injections per day since its pharmacokinetics differ from those of endogenous two-phase insulin secretion into portal circulation. To this aim, rapid-acting and long-acting (or basal) insulin analogues have been synthesized, in addition to regular insulin, in order to improve prandial and overnight glucose control, respectively [2]. The first basal insulin analogue, glargine insulin (GLA), for once-a-day administration, was designed by substituting the A21 asparagine with glycine and prolonging the B chain with two arginine residues. These changes shifted the protein’s isoelectric point, making GLA insoluble at neutral pH, leading to microcrystals being formed, from which slow drug release occurred. Nonetheless, despite the recent progress in the development of several alternative long-acting insulins, distress from multiple daily injections is still an unpleasant condition for the subjects to overcome [2,3].

Insulin pumps have been developed within this context. However, the United States Food and Drug Administration (FDA) has classified such devices as endowed with moderate-to-high risk [3]. Inhaled insulin (Exubera^®^) is a recent alternative; while associated with the fast onset of action, it lacks dosing flexibility. Moreover, relevant concerns about potential long-term pulmonary effects have inhibited its clinical use so far [3]. Oral insulin, however, is still a long sought-after alternative, despite limited peptide absorption through the intestinal mucosa and its degradation by stomach and gut proteolytic enzymes. A variety of devices designed to overcome these drawbacks have undergone thorough investigation and include permeation enhancers, mucosal patches and nanoparticles. The latter are capable of having the peptide loaded in their inner matrices, from which sustained release can be achieved over time, while their surfaces can be further functionalized with targeting moieties. This approach presents relevant and simultaneous advantages; protection from stomach-acid pH and enzymatic degradation, enhanced protein bioavailability and targeted delivery. Ideally, they should be composed of bio-adhesive, biocompatible, biodegradable and easy-to-process materials that are capable of preventing protein degradation [4,5].

Different types of nanoparticles have been purposed for the oral delivery of insulin, including those based on natural (i.e., chitosan, alginate, dextran) and synthetic (Eudragit^®^, poly(lactide), poly(lactide-co-glycolide), poly(ε-caprolactone) and poly(allylamine)) polymers [6,7,8]. Moreover, innovative composite nanocarriers, such as nanophytosomes [9] and multilayered nanoparticles, will be of interest in the near future. However, lipid nanoparticles, in particular, have shown remarkable advantages in terms of biocompatibility [6,10]. From this category, liposomes are vesicular colloidal systems with the longest history of safe clinical usage, therefore driving their candidature for the delivery of oral insulin. Accordingly, the most relevant evidence has reported that their use has resulted in significant hypoglycemia in healthy and diabetic rats [6,10]. Interestingly, improved insulin uptake may be achieved either by targeting specific sites on the intestinal epithelium with lectins grafted onto their surface [10] or via surface coating with chitosan [6]. Solid lipid nanoparticles (SLNs) are colloidal systems made up of a solid lipid matrix surrounded by surfactants. They are usually produced via the high-pressure homogenization of melted lipid emulsions [11]. Compared to liposomes, they show improved physico-chemical stability and easier production scale up [12]. Although they have also been proposed as insulin-delivery vehicles to be taken up via the gut, they are devoid of an inner aqueous compartment, unlike vesicular systems, and hydrophilic insulin is difficult to load within their lipid core [8,10]. Moreover, the high temperatures required for lipid processing may hamper the stability of thermolabile peptides.

Of the nanoparticle production methods available, coacervation works via the phase separation of a polymer aqueous solution into nanoparticles, achieved by changing environmental factors. Simple coacervation is mostly performed via polymer salting out by means of ionic interactions and pH, or temperature, shifts. Complex coacervation works through electrostatic attraction between polyelectrolyte polymers with opposite charges, leading to an insoluble complex [13]. With this method, toxic solvent usage and energy consumption are excluded, allowing the process to operate in a completely sustainable manner. Interestingly, this technique may exploit natural polymers and is also widely employed to encapsulate natural compounds [14,15]. On the other hand, so-called fatty acid coacervation provides SLNs composed of fatty acids, after the phase separation of their corresponding alkaline soaps from aqueous solution, following proton exchange. This may be achieved only upon heating the soap above its Krafft point to obtain a clear micellar solution. However, for most soaps, Krafft points are located at rather low temperatures (around 50 °C) that are compatible with most thermolabile compounds (Figure S1) [16].

For these reasons, the production of SLNs by coacervation, and obtained from synthetic soaps, has already been exploited for the oral delivery of GLA. Indeed, due to its modified amino acid sequence, this insulin analogue can undergo efficient hydrophobic ion pairing (HIP) with negatively charged surfactants, thus facilitating easy loading within SLNs and optimized release at the intestinal mucosa [17,18]. However, so-called “green SLNs” can also be formulated via coacervation using natural soaps, which are, in turn, obtained via the saponification of vegetal solid fats, such as Shea and Mango butters [19,20]. Owing to their fully natural origin, these SLNs may have promising properties for the oral delivery of insulin. Indeed, their oleic acid content can favor paracellular transport at the intestinal mucosa, thus increasing protein uptake [18,21], while polymers used as stabilizers can help bio-adhesion at the intestinal mucosa [17,21]. Moreover, the Mango and Shea unsaponifiable fraction contains antioxidant and anti-inflammatory molecules, including cinnamic acid and 3β-taraxerol, which can be beneficial for glucose control [22,23,24,25,26].

Therefore, in this experimental work, GLA has been loaded into SLNs via coacervation from Shea and Mango soaps due to HIP. The potential of such formulations as oral peptide permeation enhancers has been assessed using ex vivo tied-up-gut experiments with fluorescently labeled GLA. Moreover, in a pilot experiment, fully re-dispersible oral solid dosage forms have been obtained from SLN suspensions by means of freeze-drying and spray-drying, and they have been fully characterized in terms of their physico-chemical properties.

## 2. Materials and Methods

### 2.1. Materials

A total of 60,000–90,000 M_W_ dextran, cyclohexane, ethanol, Krebs Ringer Buffer (KRB), PVA 9000, trehalose and tetramethylrhodamine isothiocyanate (TRITC) were obtained from Sigma-Aldrich (St. Louis, MO, USA). Ethyl stearate and palmitic acid were obtained from Fluka (Buchs, Switzerland). Acetonitrile, formic acid, methanol, HCl, N,O-bis(trimethyllsilyl)trifluoroacetamide (BSTFA), pyridine, oleic acid, sodium docusate (AOT), sodium mono-hydrogen phosphate and di-hydrogen phosphate and stearic acid were obtained from Merck (Darmstadt, Germany). Glyceryl monostearate was from Goldschmidt (Essen, Germany). Glyceryl monooleate was obtained from Evonik (Essen, Germany). Lactic acid, NaOH and Shea butter were obtained from A.C.E.F. (Fiorenzuola d’Arda, Italy). 4′,6-diamidino-2-phenylindole (DAPI), H_3_PO_4_ and trifluoroacetic acid (TFA) were obtained from VWR (Radnor, PA, USA). Ethyloleate was obtained from Carlo Erba (Cornaredo, Italy). Mango butter was a kind gift from Sophim (Peyruis, France). Gohsenol^®^ was a kind gift from Harke Pharma (Mülheim an der Ruhr, Germany). Kollicoat^®^ IR was a kind gift from BASF (Ludwigshafen, Germany). Distilled water was purified using a MilliQ system (Millipore, Bedford, MA, USA). All other chemicals were of analytical grade and used without further purification.

### 2.2. Isolation of GLA from Commercial Insulin Preparations

GLA was obtained from Lantus^®^ (Sanofi-Aventis, Paris, France). Selective precipitation was performed using pH-dependent solubility variations. Nearly 3.6 mg of protein can be isolated from 1 mL of commercial Lantus^®^. Briefly, Lantus^®^ was brought to pH = 7.0 with NaOH, and the precipitated protein was then centrifuged at 2400× *g* for 5 min (Rotofix 32 centrifuge, Hettich, Tuttlingen, Germany). The supernatant was discarded, and the pellet (GLA) was washed twice with ultrapure water and dried under vacuum [17,18].

### 2.3. Fluorescent Labeling of GLA

GLA-TRITC was synthesized according to the method described by Muntoni et al. [17]. Briefly, GLA (10 mg, 0.002 mmol) was dissolved in 2.78 mL of 0.01 M HCl and added to a 3 mg/mL TRITC solution in methanol (0.9 mg, 0.002 mmol). The mixture was stirred overnight at room temperature, and then GLA-TRITC was precipitated by adding 370 μL of 0.1 M phosphate buffer pH 7.4, followed by 5 min centrifugation at 2400× *g* (Rotofix 32 centrifuge). The pellet was washed four times with the same buffer. GLA-TRITC was analyzed by HPLC. The compound was obtained as a dark red solid. The yield of GLA-TRITC derivatives was 36%, while unlabeled GLA made up the remaining 64%.

### 2.4. Ethanolic Saponification of Shea and Mango Butters

Vegetal fats were dissolved in ethanol (0.5 g/mL) at 50 °C. NaOH was pre-dissolved in 3 mL of ethanol at 60 °C and then introduced into the fat solution under continuous stirring at appropriate dilution (21.33 mg/mL). The saponification process lasted 6–8 h under stirring at 50 °C. In the case of Shea butter, a 50% excess with respect to the theoretical saponification index was used to bring the reaction to completeness. Ethanol was then evaporated under nitrogen flow, and the obtained natural soaps were maintained for further drying under vacuum for 2 days before use.

### 2.5. Determination of the Composition of Shea and Mango Soaps

Gas chromatography coupled with mass spectrometry (GC-MS) was used to qualitatively and quantitatively characterize the lipid composition of the Mango and Shea soaps. The sample preparation used was the same as in our previous work [19]. A weighed amount (1 mg) of the samples was derivatized with BSTFA to obtain the trimethylsilyl derivatives. Specifically, 80 μL pyridine and 120 μL BSTFA were added to 1 mg soap, and the solution was then heated to 60 °C in a water bath for 30 min. The samples obtained were subsequently analyzed by GC-MS (an Agilent 6890 GC unit coupled to an Agilent 5973 MSD, Agilent, Little Falls, DE, USA) according to the method reported by Bozza et al., 2024 [19]. The compounds were identified by comparing their mass spectra with those reported in the literature and in the available databases (Wiley and Nist) and confirmed by co-injection of the commercial reference standards, where available. Semi-quantification was carried out with the external standard calibration method in single-ion monitoring (SIM) mode, with a target ion being selected for each compound: 313 *m*/*z* for palmitic acid, 101 *m*/*z* for oleic acid ethyl ester, 88 *m*/*z* for stearic acid ethyl ester, 339 *m*/*z* for oleic acid and 341 *m*/*z* for stearic acid. Each compound was quantified using the single-point quantification method, in which the peak area of the reference standard at a known concentration was compared with the peak area of the compound contained in the extract. For the quantification of the fatty acid ethyl esters, a weighed amount of the soaps/standards was dissolved in cyclohexane. Data were processed using GCMS Solution v4.30 software (Shimadzu, Tokyo, Japan).

### 2.6. GLA HIP

GLA (3.6 mg/mL, 0.00034 mmol) was dissolved in 560 µL of 0.1 M HCl. Subsequently, 0.0028 mmol of AOT was added with an insulin–surfactant ratio of 1:8 [17,18]. Indeed, GLA contains 8 ionizable basic groups—2 amino terminals, 3 arginine, 1 lysine and 2 histidine—that can contribute to the electrostatic interactions with the surfactant. Briefly, 277 µL of AOT (4.5 mg/mL) was added, precipitating the GLA-AOT ion pair. The suspension was then centrifuged at 14,300× *g* for 10 min (MPW55, Medical Instruments, San Lazzaro di Savena, Italy). The pellet was dissolved in 160 µL of ethanol (12 mg/mL). The same process was employed with fluorescently labeled GLA-TRITC, which was used for ex vivo tied-up-gut studies [17,18].

### 2.7. Formulation of GLA-Loaded SLNs

SLNs were prepared via the fatty acid coacervation method [17,18]. Briefly, Mango and Shea soaps (120 mg, 1%) were dispersed in water, and the mixture was then heated to 70 °C under stirring (300 rpm) to obtain a clear micellar solution. The temperature was increased to 50 °C, and then 1 M lactic acid (400 μL), mixed with a 10% PVA 9000 solution (1 mL, 1%), was added dropwise as the coacervating solution, until complete fatty acid precipitation occurred. Finally, the obtained suspension was cooled in an ultrasonic bath (Transsonic 660/H, Elma Schmidbauer GmbH, Singen, Germany). A heating and cooling cycle was then performed under stirring. SLNs were allowed to fully precipitate overnight and then filtered with paper, to remove aggregates. Peptide loading was performed as follows: two mL of green SLNs were heated up to 80 °C; the temperature was then brought to 55 °C, and either the GLA or GLA-TRITC ion pairs in ethanol (12 mg/mL) were loaded into the SLNs, up to a final peptide concentration of 0.5–1 mg/mL; the suspension was cooled to room temperature.

### 2.8. Physico-Chemical Characterization of Suspensions

The mean particle diameter and polydispersity index (PDI) of SLNs were measured using the DLS technique (90 Plus, Brookhaven, NY, USA). Measurements were performed at an angle of 90° at 25 °C. The homogeneity of SLN suspensions was preliminarily checked by OM (DM2500, Leica Microsystems, Wetzlar, Germany).

Drug recovery (%), defined as the ratio between actual and theoretical peptide concentration in SLN suspensions, and drug entrapment efficiency (EE%), defined as the ratio between the entrapped lipid and total peptide in suspension, were determined after lipid matrix separation by ultracentrifugation.

To this aim, two samples containing 250 μL of SLN suspension were diluted with 250 μL of a 30% water solution of 60,000–90,000 M_W_ dextran and ultra-centrifuged at 62,000× *g* for 15 min (Allegra^®^ 64 R centrifuge, Beckman Coulter, Palo Alto, CA, USA). In this way, the unloaded GLA-AOT was separated into the supernatant. In separate experiments, the obtained lipid pellets were washed to remove the peptide adsorbed onto the SLN surface. These washings were performed with either 250 μL of 0.1 M HCl or 0.05 M phosphate buffer, pH 8.0, in order to dissolve GLA or to displace the GLA-AOT ion pair, respectively. Subsequently, 250 μL of a 30% water solution of 60,000–90,000 M_W_ dextran were added to each of the samples, which were then ultra-centrifuged at 62,000× *g* for 15 min (Allegra^®^ 64 R centrifuge). In order to extract insulin, lipid pellets were dissolved in 150 μL of ethanol, diluted with 100 μL of ultrapure water and ultra-centrifuged at 62,000× *g* for 5 min (Allegra^®^ 64 R centrifuge). All of the dextran gradient ultracentrifugation steps (used to favor lipid pellet outcrop) were conducted in plastic-ware. The samples obtained (supernatant, washings and pellet extractions) were analyzed by high-pressure liquid chromatography (HPLC) [17]. Prior to HPLC injection, the supernatant and washings were diluted 4-fold in ethanol and centrifuged at 14,300× *g* for 10 min (MPW55), in order to remove 60,000–90,000 M_W_ dextran.

The % recovery of the peptide loaded into the SLNs was calculated as the ratio between the sum of the amounts recovered in the supernatant, washings and pellet extractions vs. GLA weight, while the EE% was calculated as the ratio between the amount in the pellet extraction and the sum of the amounts in the supernatant, washings and pellet extractions, for each condition under study.

### 2.9. Formulation of Oral Solid Dosage Forms

#### 2.9.1. Freeze-Drying of SLN Suspensions

Pilot freeze-drying experiments were performed to optimize the ratio between GLA-AOT-loaded SLNs and a cryoprotectant (cryo) solution (7% trehalose, 7% Gohsenol^®^ and 6% Kollicoat^®^ IR), which has already been used in the literature [18], up to various ratios, prior to lyophilization (Christ, Alpha 1-2 LD, Osterode am Harz, Germany).

#### 2.9.2. Spray-Drying of SLN Suspensions

The optimized cryo–SLN ratios, selected in the pilot freeze-drying experiments, were then employed for spray-drying experiments, which allow for better process scale-up compared to freeze-drying. Green SLNs loaded with 1 mg/mL GLA-AOT, both Mango and Shea, were used. Immediately after preparation, SLNs were diluted 2:1 in the cryo solution (final GLA-AOT concentration of 0.5 mg/mL). The spray-drying (SD-2L, ZZKeda Machinery and Instrument Equipment Co., Ltd., Zhengzhou, China) conditions were as follows: Inlet T = 60 °C; Outlet T = 40 °C; Air fan = 100%; Pump Rate = 12%; Nozzle Ø = 0.75 mm; pressure (mbar) = 1500; Cleaning pin = 6 s.

#### 2.9.3. Formulation of Tablets from Spray-Dried Powders

Re-dispersible powders, produced via spray-drying, were used to form mini tablets (3 mm diameter, 60 mg weight) by direct compression (Ek0d, Korsh AG, Berlin, Germany). An effervescent mixture (3.5 mg citric acid and 3.5 mg sodium carbonate) was added to each tablet as the disintegrant [18].

### 2.10. Physico-Chemical Characterization of Solid Oral Dosage Forms

Solid oral dosage forms (powders and tablets) were weighed, and 1 mL of ultrapure water was then added to each sample to allow for re-suspension under magnetic stirring. The time needed for re-suspension was measured, as well as the homogeneity of the obtained suspension, and its mean size and PDI by DLS (90 Plus). In the case of the tablets, the re-suspension time was determined in the presence and in the absence of the effervescent disintegrant mixture. Moreover, the European Union (EU) Pharmacopoeia disaggregation assay was performed on 6 tablets obtained by spray-drying from SLNs loaded with GLA-AOT. The disaggregation time of the tablets was determined only in the presence of the effervescent disintegrant mixture.

The recovery (%) of the GLA-AOT loaded into the SLN suspensions, obtained after the redispersion of freeze-dried and spray-dried powders, was also measured as the ratio between actual drug concentration and the theoretical amount of GLA insulin-AOT, based on the estimated dry weight of freeze-dried samples. Briefly, 50 μL of re-dispersed suspension was dissolved in 100 μL ethanol, the lipid was precipitated by adding 50 μL of ultrapure water, the obtained mixture was centrifuged at 14,300× *g* for 15 min (MPW55 centrifuge) and the supernatant was injected into the HPLC system.

### 2.11. Pilot Non-Everted Tied-Up-Gut Experiments

An ex vivo absorption evaluation was carried out using permeation measurements in excised rat small intestine, as described by Muntoni et al. [17]. Male Wistar rats (250 g) were anesthetized with isoflurane, sacrificed and exsanguinated, according to experimental protocol number 56105.N.WSP, as approved by the Italian Ministry of Health on 14 July 2023. Freshly excised rat duodenum, jejunum and ileum tissue was washed with KRB buffer and cut into pieces of 4–5 cm. GLA-TRITC-AOT loaded SLNs (200 μL) were syringed into intestinal sacs, and the filled tissues were incubated in oxygenated KRB (10 mL) at 37 °C with smooth shaking. Samples of KRB (500 μL) were withdrawn from the serosal side at fixed time intervals up to 120 min and replaced with fresh buffer. Tests were performed in replicate for each formulation under study, using guts from 2 different rats.

The labeled peptide concentration in the incubation buffer was quantified by fluorescence measurements using a multilabel plate reader (Victor3 1420, Perkin Elmer, Waltham, MA, USA): λ_exc_ = 540 nm, λ_em_ = 575 nm. Quantitation was performed using a calibration curve (0.084–5.0 μg/mL) (R^2^ > 0.99).

At the end of the experiments, tissues were washed with normal saline solution (0.9% *w*/*v* NaCl). Small intestinal tissue sections were embedded in Optimal Cutting Temperature (OCT) compound for cryostat sections at controlled temperature (−15 ± 1 °C). Five-micrometer-thick sections were prepared using a cryostat microtome (Reichert-Jung/Leica, Frigocut 2800 N), labeled with DAPI staining and mounted onto slide glasses. Tissue sections were visualized and imaged using a DM2500 fluorescence microscope.

### 2.12. HPLC Analysis

HPLC analysis was performed using a YL9110 Quaternary Pump, equipped with a Shimadzu RF-20 fluorescence detector (Shimadzu, Tokyo, Japan), linked to Clarity software for data analysis (Yang Lin, Anyang, Republic of Korea, version 3.0.4.444). The column was a Zorbax XDB C8 12.5 × 0.46 cm (Agilent Technologies, Santa Clara, CA, USA). The eluent was composed of 0.1% TFA in MilliQ water (A) and acetonitrile (C), eluted with a gradient analysis (min 0: 100% A, 0% C; min 15: 40% A, 60% C; min 20: 40% A, 60% C; min 22: 100% A, 0% C; min 23: 100% A, 0% C). The flow rate was set at 1 mL/min. A photodiode array (PDA) was set at λ = 220 nm for Glargine insulin, while GLA-TRITC was identified at λ = 540 nm and quantified at λ = 220 nm. Retention times were 11 min for Glargine insulin and 12.0, 13.5 and 13.8 min for GLA-TRITC derivatives. GLA (R^2^: 0.9999); GLA-TRITC (R^2^: 0.9979).

### 2.13. Statistical Analysis

Results are reported as mean ± standard error of the mean (SEM).

In technological experiments, statistical analyses were designed to compare all liquid SLNs suspensions against each other in order to evaluate the influence of each formulation parameter. In the case of solid dosage forms, however, the Shea and Mango series were compared separately in order to evaluate the influence of the selected drying process. Either one-way ANOVA, followed by Bonferroni’s multiple comparison test, or two-tailed unpaired *t*-tests were performed using the Graphpad Prism 5.0 software (Graphpad Software, San Diego, CA, USA, 2016), depending on the number of experimental groups to be compared (either > or =2, respectively).

The tied-gut experiments on intestinal permeation were conducted as a pilot study. Accordingly, the sample size was limited to two rats, in order to use the minimum number of animals required to obtain preliminary insights into the behavior of the different groups. For statistical analysis, the experimental design included one independent variable (gut permeation) and three groups (two loaded SLNs and the free labeled peptide). Group means were compared using one-way ANOVA, followed by Bonferroni’s multiple comparison test, to identify significant differences between selected pairs. Statistical analysis was performed using Graphpad Prism 5.0 software.

## 3. Results

### 3.1. Physico-Chemical Characterization

#### 3.1.1. SLN Suspensions

**Figure 1:**
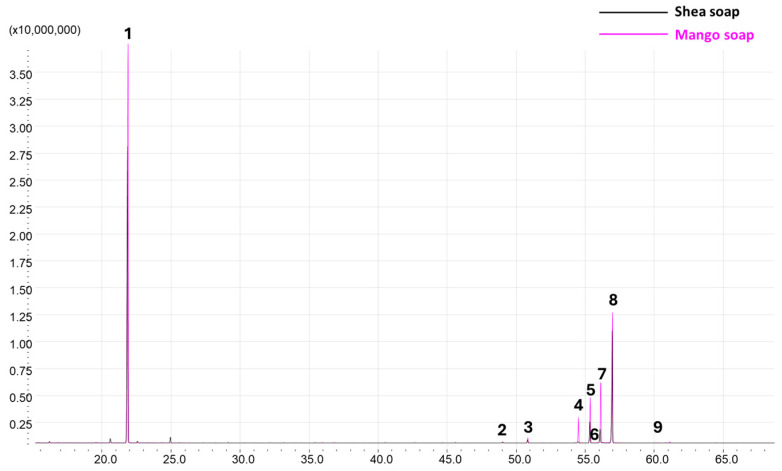
Gas chromatography coupled to mass spectrometry (GC-MS) profiles of Shea and Mango soaps after derivatization. Peak numbers refer to Table 1.

In previous studies, the stability of green SLNs was reported to be negatively affected by the presence of residual monoglycerides in the starting natural soaps [19]. Given that stability is a key issue for such formulations, in this experimental work, soaps were obtained via the ethanolic saponification of Mango and Shea butters to minimize residual monoglycerides as it is a more efficient hydrolysis process. Indeed, although monoglycerides (i.e., glyceryl monostearate, glyceryl monooleate) were no longer present in the soaps, ethyl esters (ethylstearate—stearic acid ethyl ester; ethyloleate—oleic acid ethyl ester) were detected, likely due to trans-esterification occurring during the saponification process. Moreover, oleic acid was present in lower amounts in Shea soap when using this saponification technique compared with previous reports [19], while linoleic and arachidic acids were below the limit of quantification in both Mango and Shea (Table 1 and Figure 1).

GLA and its fluorescent derivative GLA-TRITC were loaded into green SLN suspensions through HIP at 0.5 mg/mL. In this study, % recovery and EE% were determined in the same sample. The effective encapsulation of GLA in the lipid core of SLNs was assessed using specific purification steps. To this aim, SLNs first underwent dextran gradient ultracentrifugation in order to separate the lipid matrix (pellet) from the supernatant, which contained the unloaded GLA-AOT. The pellet was then washed under two different conditions: 0.01 N HCl and phosphate buffer pH = 8.0. Indeed, such aqueous solutions cannot solubilize the lipid matrix but are capable of dissolving the GLA adsorbed onto the SLN surface and of displacing GLA-AOT HIP, respectively (Figure S2) [17,18].

Experimental data showed high variability, which prevented statistically significant differences from being observed. This might be ascribed to the aforementioned complex procedure needed to only isolate the GLA that is effectively bound to the SLN lipid matrix. However, in general, Shea SLNs showed a higher trend for GLA recovery compared to Mango SLNs, but also a lower EE% after washing under the different conditions. With regards to GLA-TRITC-AOT-loaded SLNs, data were even more variable. It is worth noting that GLA-TRITC-AOT loading might be affected by the co-existence of unlabeled GLA in the in-house synthesized fluorescent peptide. Indeed, it was not possible to separate pure labeled GLA from the unlabeled protein, and the two compounds might compete with the AOT pairing agent with different affinity. However, labeled GLA is not thought to be used for therapeutic delivery, but only to investigate gut permeation in ex vivo models.

Finally, the presence of ethyl esters, mentioned above, had no relevant effect on the stability of GLA-loaded SLNs, since mean particle size did not increase over time (Table 2, Figures S3 and S4).

#### 3.1.2. Solid Dosage Forms

Solid oral dosage forms (re-dispersible powders, tablets) were obtained from SLN suspensions by means of freeze-drying and spray-drying techniques. To this aim, SLN suspensions loaded with 1 mg/mL GLA-AOT were preliminarily diluted in a cryo solution, in order to minimize drug dilution during the process.

Freeze-drying was performed, in the first instance, on small samples to optimize the ratio between SLNs and cryo. Only Shea SLNs were used in these experiments, which had the aim of improving the conditions for achieving fully re-dispersible powders. A total of 1 mL Shea SLNs GLA-AOT was alternatively diluted with 1 mL cryo solution (1:1); 0.5 mL cryo solution (2:1); 0.25 mL cryo solution (4:1) and 0.1 mL cryo solution (10:1). The final volume was brought to 2 mL with ultrapure water, meaning that the GLA-AOT concentration for each cryo–SLN mixture before freeze-drying was 0.5 mg/mL.

In solid dosage forms, drug recovery was calculated simply via direct extraction from the suspension obtained after redispersion. Interestingly, drug recovery in the conventional SLN suspension loaded with 0.5 mg/mL GLA-AOT (Table 2) and the SLN loaded with 1 mg/mL peptide, used as an intermediate to produce solid dosage forms, were comparable (Table 3). Moreover, good recovery retention was obtained with the 2:1, 4:1 and 10:1 SLNs/cryo volume ratios, compared to the suspension. However, lower recovery and longer re-dispersion times were observed when adopting the 1:1 ratio. Since the results from the 10:1 ratio re-dispersion were not fully reproducible, the 2:1 and 4:1 ratios were selected for further spray-drying experiments in scale-up mode (Table 3, Figures S5–S7).

The spray-drying technology can be scaled up more easily than freeze-drying, allowing for further processing of the re-dispersible powders, in this case, formulation into tablets via direct compression. Nonetheless, the spray-dried powder from the 4:1 SLN/cryo ratio, attempted only with Shea SLNs, was difficult to process with direct compression as it was too soft. Thus, only the 2:1 ratio yielded spray-dried powders, from both Mango and Shea SLNs, that were suitable for formulation into tablets. The yield of the spray-drying process was 80% for Shea and 67% for Mango SLNs in the collecting vessel, with negligible residue in the cyclone. SLN suspensions loaded with 1 mg/mL GLA-AOT were used as intermediates for spray-drying. In suspension, Shea SLNs showed higher recovery (and particle size) than Mango SLNs. This, in turn, was reflected in a slight decrease in peptide content in Mango-based tablets compared to Shea ones. Nonetheless, the differences in tablet recovery were not statistically significant, likely due to a lack of homogeneity in the powders used to produce them. Importantly, the re-dispersion capability of the powders, in terms of mean particle size, was not negatively affected by direct compression. Indeed, a homogenous colloidal system was obtained after redispersion, without an increase in mean particle size. However, the re-dispersion time largely increased after compression, shifting from 6 to over 30 min. Therefore, an effervescent mixture (citric acid + NaHCO_3_) was employed to decrease the re-dispersion and disaggregation times, down to nearly 15 min (Table 4, Figures S5–S7).

### 3.2. Ex Vivo Studies

**Figure 2:**
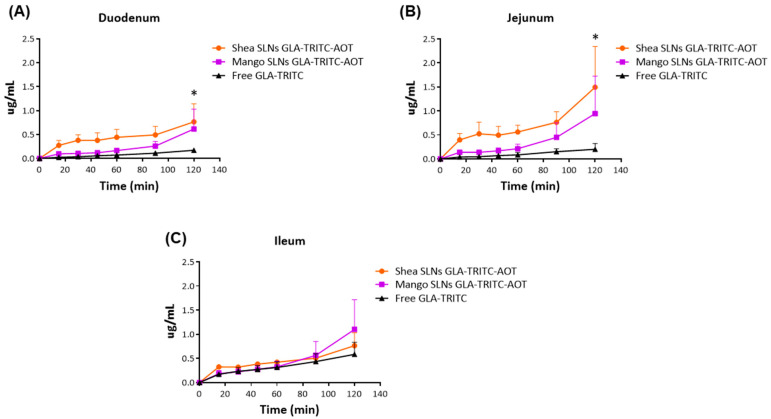
(**A**) Duodenum, (**B**) jejunum and (**C**) ileum permeation of green SLNs loaded with GLA-TRITC-AOT and free GLA-TRITC (positive control) in rat guts. Statistical analysis. One-way ANOVA, followed by Bonferroni’s multiple comparison test. * *p* < 0.05: Shea SLNs GLA-TRITC-AOT vs. free GLA-TRITC total curve in duodenum and jejunum. Abbreviations: AOT: sodium docusate; GLA: glargine insulin; SEM: standard error of the mean; SLNs: solid lipid nanoparticles; TRITC: tetramethylrhodamine isothiocyanate.

To explore the ability of green SLNs to enhance the permeation of the loaded GLA, a pilot ex vivo tied-up-gut study was conducted. To this aim, GLA-TRITC-AOT loaded SLNs were used. GLA-TRITC uptake within duodenum and jejunum gut segments was improved by loading within SLNs, with Shea SLNs providing a significantly higher permeation curve than free labeled peptide (positive control) (* *p* < 0.05, one-way ANOVA) (Figure 2).

**Figure 3:**
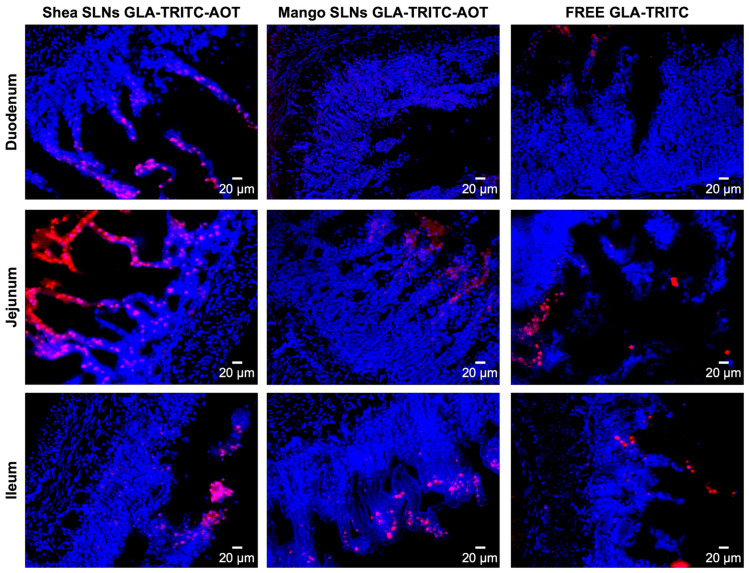
Fluorescence microscopy of different gut sections after administration of green SLNs loaded with GLA-TRITC-AOT and free GLA-TRITC. Blue: nuclei labeled with DAPI. Red: labeled GLA. Magnification 10×. Scale bar: 20 μm. Abbreviations: GLA: glargine insulin; AOT: sodium docusate; DAPI: 4′,6-diamidino-2-phenylindole dihydrochloride; SLNs: solid lipid nanoparticles; TRITC: tetramethylrhodamine isothiocyanate.

Fluorescence microscopy of histological gut sections allowed the association between TRITC-GLA from SLNs and gut villi to be assessed. Despite only being qualitative, this evidence showed that Shea SLNs had greater fluorescence intensity than Mango SLNs (Figure 3).

## 4. Discussion

If effective, the oral route would be the optimal method for insulin delivery, as the peptide undergoing a first-pass effect decreases the hepatic glucose output while limiting peripheral hyper-insulinemia and related adverse effects [27]. However, oral insulin still poses quite the challenge, due to peptide degradation and limited absorption in the gut. To counter this, SLNs can enhance protein uptake by the gut, through both the intestinal mucosa and Peyer’s patches [28]. Nonetheless, insulin loading in SLNs has been hampered so far by its hydrophilic and thermolabile nature. In particular, the latter feature precludes the use of the most widespread SLN formulation techniques, which operate at high temperatures, while solvent-based processes require extensive purification steps to overcome the intrinsic toxicity of residual solvents [29]. HIP has emerged as a suitable method to improve peptide loading in lipid-based matrices, while fatty acid coacervation techniques allow the operating temperature to be minimized by proceeding in solvent-free mode [17,18]. Furthermore, green SLNs may enhance gut-wall permeability by exploiting the intrinsic components of a naturally derived innovative matrix. Interestingly, the ethanolic saponification of the starting butters allows the presence of residual monoglycerides in the soap, which are detrimental to the coacervation process, to be minimized [19]. However, it induces the formation of fatty acid ethyl esters, likely due to a trans-esterification phenomenon, which seem not to affect the physico-chemical stability of SLNs to a remarkable extent (Table 2).

GLA is insoluble at neutral pH because its modified amino acid sequence causes it to have a different isoelectric point to human insulin. In humans, this causes the formation of peptide microcrystals after subcutaneous injection, from which slow GLA release may be achieved, which is suitable to guarantee basal insulin levels [30]. On the other hand, from a pure physico-chemical standpoint, GLA is endowed with superior HIP capability compared to human insulin. This, in turn, favors a strong association between GLA-AOT and the green SLN lipid matrix, whereas quick release in the intestinal lumen would expose the unbound peptide to digestive enzymes, with poor gut uptake. Accordingly, previous evidence has shown that GLA can be taken up through the gut mucosa, likely exploiting the paracellular route, after peptide displacement from HIP by the mucin [17,18]. Advantageously, the oleic acid content of green SLNs should promote such a mechanism by altering the tight junctions at the intestinal mucosa. Via this mechanism, green SLNs show the potential to act as smart delivery systems for GLA. Indeed, they are anticipated to protect the peptide from enzymatic degradation in the gastrointestinal lumen once effectively loaded within the lipid matrix while improving intestinal uptake and oral bioavailability through specific biological interactions with the gut wall, where peptide release should specifically occur [14,31]. Moreover, free fatty acids, such as those constituting the lipid matrix of green SLNs, might favor glucose control in diabetes. Indeed, dietary fatty acids are taken up with high efficiency by the gut through specific receptors, such as FATP4, a member of a large family of fatty acid transport proteins (FATPs) that is expressed at high levels on the apical side of mature enterocytes in the small intestine. Then, absorbed fatty acids may specifically trigger GPR40, a G-protein-coupled receptor abundantly expressed in pancreatic β cells, which, in turn, can amplify glucose-stimulated insulin secretion and, thus, has been a subject of investigation for innovative anti-diabetic drugs [32,33,34].

In this context, the permeability of GLA-TRITC across rat small intestine was preliminarily investigated ex vivo with the non-everted intestinal sac method. Indeed, despite the absence of blood flow and a nervous system, this model is widely employed to evaluate passive compound uptake because it is characterized by the mucus layer, transport proteins and drug metabolism [35,36]. In these experiments, green SLNs favored the uptake of the loaded fluorescent peptide into the duodenum and jejunum (Figure 2). The lack of significant differences in absorption through the ileum appears to be due to the increased permeation of the free peptide in this gut section compared with the duodenum and jejunum, rather than to lower absorption of GLA-TRITC loaded SLNs. Accordingly, it has been observed that, despite being lower than in the intestinal lumen, the protease content varies in rats across the mucosa of the different intestinal regions, with higher levels being present in the upper small intestine compared to the descending small intestine [37]. Thus, enhanced free GLA-TRITC permeation may result from lower protease activity in the ileum compared with the upper small intestine, leading to reduced proteolytic degradation of the free peptide and, consequently, greater translocation across the intestinal wall. Moreover, M cells in Peyer’s patches, particularly abundant in the ileum, are considered key sites for the pinocytosis of large molecules, enabling peptide uptake into the lymphatic system [37]. This anatomical feature may also explain the greater free peptide permeation in the ileum compared to the duodenum and jejunum.

Moreover, in the perspective of potential translation to humans, solid oral dosage forms were obtained from SLN suspensions, since they may improve patient compliance and minimize physico-chemical and microbiological stability concerns. Freeze-drying and spray-drying can be alternatively used to this aim, with the former limited by lower scale-up capability and the latter by potential degradation of thermolabile compounds, particularly peptides [18]. However, in both cases, the use of a cryo mixture was mandatory. The primary reason was to increase the solid/liquid ratio, since a higher content of liquids (i.e., unsaturated fatty acids from SLNs) would lead to a sticky and soft powder, which is unsuitable for further processing by direct compression. In addition, the cryo can act as a spacer between nanoparticles, improving the hydration ability of their external layer and thus facilitating the re-dispersion process. In this way, when put in contact with water under simple magnetic stirring, the obtained powders should be able to reproduce the original suspension, without macroscopic aggregates, while maintaining drug content. The SLN/cryo ratio was set up by preliminary freeze-drying experiments (Table 3), while the scaled-up production of re-dispersible powders and tablets was achieved by spray-drying at an optimized 2:1 ratio (Table 4). Moreover, in order to limit peptide dilution in the solid form, SLN suspensions with higher peptide content (1 mg/mL) ratios were employed as a smart intermediate. The combined use of sugars and polymers as the cryo mixture is well-known to improve the re-dispersion capability of powders [18]. It is worth noting that trehalose, despite being a disaccharide, is reported not to cause fast glycemic increase [38,39]. Compared with previous reports [18], green SLN led to an increase in the SLN/cryo ratio, which, in turn, results in a higher peptide content in the final solid dosage form. However, longer re-dispersion times are obtained with the lowest SLN/cryo ratio (1:1), while less reproducible re-dispersion is achieved with the highest one (10:1). Powders obtained with intermediate SLN/cryo ratios (4:1 and 2:1), however, are characterized by shorter re-dispersion times and good drug-recovery retention. Interestingly, no significant difference was detected in protein recovery between spray-drying and freeze-drying processes for Shea-based formulations, thus allowing thermal degradation to be excluded from the operative conditions adopted.

The direct compression of the re-dispersible powders poses further concerns. The polymers of the cryo mixture (Kollicoat^®^ IR and Gohsenol^®^) are widely used as excipients for the direct compression of prolonged release tablets. Thus, they delay disintegration times excessively for our purposes. Furthermore, powder from a 4:1 SLN/cryo ratio is too soft for direct compression, whereas a 2:1 ratio is the best compromise, since it allows for good compressibility and optimized GLA recovery. Improved tablet re-suspension and disaggregation times were obtained with a small quantity of an effervescent mixture (citric acid + NaHCO_3_).

## 5. Conclusions

In this study, GLA has been loaded into green SLNs via coacervation from natural soaps using HIP. Preliminary ex vivo studies have demonstrated that green SLNs can enhance the permeation of the loaded GLA through the duodenum and jejunum, likely due to the unsaturated fatty acid content of the lipid matrix. Furthermore, a re-dispersible powder composition has been optimized by freeze-drying the SLN suspension and scaled up by spray-drying; this latter process is suitable for the formulation of solid oral dosage forms such as granulates and tablets. Although our findings are promising, they remain preliminary, particularly in terms of biological evaluations, making further in vivo studies a necessity. Firstly, pharmacokinetics assays would allow the timing and extent of GLA uptake by the gut, with respect to the administered dose of either liquid or solid dosage forms, to be understood. Secondly, using this pharmacokinetic study as a basis, dose–response glucose-lowering efficacy should be determined in diabetic animal models in order to confirm the therapeutic potential of this approach for diabetes management.

## Figures and Tables

**Table 1:** Lipid composition and percentage in Mango and Shea soaps. Semi-quantitative results of the selected compounds (in bold) were obtained via quantification using single-point calibration in GC-MS-SIM, as described in Section 2.5. Results are expressed as a percentage in dry soap. The rsd% is reported in brackets. Abbreviations: GC-MS-SIM: gas chromatography coupled to mass spectrometry in single-ion monitoring mode; M_W_: molecular weight; rsd%: percent relative standard deviation; TMS: trimethylsilane.

Peak N°	Retention Time (min)	Compound Name	M_W_ **	Shea Soap (%)	Mango Soap (%)
1	21.844	Glycerol 3TMS *	308		
2	48.998	Palmitic acid ethyl ester	284		
3	50.803	Palmitic acid TMS *	328	3.9% (rsd%: 8.1%)	8.2% (rsd%: 16.0%)
4	54.448	Oleic acid ethyl ester *	310	0.8% (rsd%: 8.5%)	13.6% (rsd%: 23.9%)
5	55.318	Stearic acid ethyl ester *	312	4.2% (rsd%: 13.4%)	9.6% (rsd%: 18.3%)
6	55.860	Linoleic acid TMS *	352		
7	56.073	Oleic acid TMS *	354	3.9% (rsd% 46.8%) *	32.3% (rsd%: 6.6%)
8	56.943	Stearic acid TMS *	356	12.9% (rsd%: 3.9%)	27.1% (rsd%: 23.1%)
9	61.179	Arachidic acid ethyl ester	340		

* Compounds were confirmed by the co-injection of the authentic reference standards. ** M_W_ for the derivatized compounds corresponds to the TMS derivative.

**Table 2:** Physical characterization, stability at 4 °C for T = 30 days and drug loading assessment of SLNs loaded with 0.5 mg/mL of either GLA-AOT or GLA-TRITC-AOT. Abbreviations: AOT: sodium docusate; GLA: glargine insulin; PDI: polydispersity index; SLNs: solid lipid nanoparticles; TRITC: tetramethylrhodamine isothiocyanate.

	T0					T = 30 Days	
	Mean Size (nm)	PDI	Recovery (%)	EE%		Mean Size (nm)	PDI
				pH 2.0	pH 8.0		
Shea SLNs GLA-AOT	325.9 ± 9.6	0.198 ± 0.021	87.3 ± 8.7	38.8 ± 14.7	12.3 ± 2.3	348.6 ± 15.9	0.076 ± 0.054
Mango SLNs GLA-AOT	302.8 ± 4.7	0.084 ± 0.028	61.3 ± 17.2	70.4 ± 19.0	63.0 ± 24.4	279.2 ± 5.4 ** #	0.065 ± 0.021
Shea SLNs GLA-TRITC-AOT	408.4 ± 41.8 *	0.147 ± 0.071	95.9 ± 14.0 */*90.2 ± 14.9* **	78.0 ± 2.4 */*66.4 ± 5.7* **	39.3 ± 1.2 */*30.2 ± 4.2* **	453.9 ± 27.6 °°°	0.087 ± 0.042
Mango SLNs GLA-TRITC-AOT	388.1 ± 2.7	0.067 ± 0.038	65.3 ± 23.2 */*34.9 ± 11.6* **	49.7 ± 6.3 */*87.1 ± 0.7* **	33.6 ± 9.9 */*79.2 ± 10.1* **	301.0 ± 0.7 ççç ###	0.095 ± 0.032

Statistical analysis: one-way ANOVA, followed by Bonferroni’s multiple comparison test. Mean size: * Mango SLNs GLA-AOT vs. Shea SLNs GLA-TRITC-AOT. Mean size after 30 days: ** Shea SLNs GLA-AOT vs. Shea SLNs GLA-TRITC-AOT; °°° Mango SLNs GLA-AOT vs. Shea SLNs GLA-TRITC-AOT; ççç Shea SLNs GLA-TRITC-AOT vs. Mango SLNs GLA-TRITC-AOT. Two-tailed unpaired *t*-test. Mean size just prepared vs. 30 days: ### Mango SLNs GLA-TRITC-AOT; # Mango SLNs GLA-AOT. Values in italics refer to unlabeled GLA in the GLA/GLA-TRITC mixture.

**Table 3:** Characterization of powders obtained by freeze-drying. Abbreviations: Cryo: cryoprotectants; PDI: polydispersity index; SLNs: solid lipid nanoparticles.

SLNs/Cryo Mixture Ratio	Redispersion Time (min)	Recovery (%)	Mean Size (nm)	PDI
suspension	-	86.0 ± 0.4	455.5 ± 5.2	0.149 ± 0.044
1:1	40 ± 3	45.8 ± 0.9 ***	385.9 ± 27.7	0.183 ± 0.045
2:1	6 ± 1 ***	67.4 ± 2.6 * °	404.8 ± 9.3	0.147 ± 0.047
4:1	3 ± 1 ***	72.6 ± 0.7 °°	448.2 ± 9.1	0.024 ± 0.053
10:1	1.5 ± 0.2 ***	71	384.0 ± 14.1	0.213 ± 0.048

Statistical analysis: one-way ANOVA, followed by Bonferroni’s multiple comparison test. Redispersion time: *** 1:1 vs. all other formulations. Recovery: *** suspension vs. 1:1; * suspension vs. 2:1; ° 1:1 vs. 2:1; °° 1:1 vs. 4:1. For the 10:1 ratio, re-dispersion was not reproducible: single recovery analysis performed.

**Table 4:** Characterization of tablets obtained by spray-drying. Abbreviations: Eff.: effervescent mixture; cryo: cryoprotectants; PDI: polydispersity index; SLNs: solid lipid nanoparticles; ND: not determined.

	Shea				Mango		
	Suspension	Plain Tablet	Plain Tablet	Tablet + Eff.	Suspension	Plain Tablet	Tablet + Eff.
SLNs/cryo ratio	-	4:1	2:1	2:1	-	2:1	2:1
Mean size (nm)	455.5 ± 5.2	412.1 ± 7.9	327.8 ± 27.2 **	333.6 ± 11.2 **	280.7 ± 2.6 °°°	272.7 ± 17.6	290.5 ± 7.7
PDI	0.149 ± 0.044	0.174 ± 0.031	0.108 ± 0.053	0.142 ± 0.018	0.073 ± 0.057	0.033 ± 0.022	0.140 ± 0.019
Recovery (%)	86.0 ± 0.4	66.0	67.8 ± 1.2	71.1 ± 11.5	69.7 ± 2.0 *	46.7 ± 13.5	49.9± 6.6
Redispersion time (min)	-	ND	30.0 ± 3.6	16.0 ± 2.0 *	-	36.0 ± 4.0	18.0 ± 1.5 °
Disaggregation time (min)	-	ND	ND	15.3 ± 0.5	-	ND	14.1 ± 1.7

Statistical analysis. One-way ANOVA, followed by Bonferroni’s multiple comparison test. Mean size: Shea series: ** Suspension vs. tablet formulations. Two-tailed unpaired *t*-test. Mean size: °°° Shea suspension vs. Mango suspension. Recovery: * Shea suspension vs. Mango suspension. Redispersion time: Shea series: * Plain Tablet vs. Tablet + Eff.; Mango series: ° Plain Tablet vs. Tablet + Eff. For the 4:1 ratio, direct compression was difficult since the powder was too soft; single recovery analysis was performed.

## Data Availability

The data presented in this study are available on request from the corresponding author.

## Supplementary Materials

The following supporting information can be downloaded at: https://www.mdpi.com/article/10.3390/pharmaceutics17101261/s1, Abbreviation list: AOT: sodium docusate; EE%: % entrapment efficiency; Eff.: effervescent mixture; GLA: glargine insulin; SLNs: solid lipid nanoparticles; TRITC: tetramethylrhodamine isothiocyanate. Figure S1: Scheme of fatty acid coacervation. Figure S2: Scheme of extraction procedure of GLA from SLNs. Figure S3: Recovery and EE% of GLA-AOT and GLA-TRITC-AOT loaded SLNs (from Table 2). Figure S4: Mean size of GLA-AOT and GLA-TRITC-AOT loaded SLNs (from Table 2). Statistical analysis: one-way ANOVA, followed by Bonferroni’s multiple comparison test. Left panel: * Mango SLNs GLA-AOT vs. Shea SLNs GLA-TRITC-AOT; right panel: ** Shea SLNs GLA-AOT vs. Shea SLNs GLA-TRITC-AOT; °°° Mango SLNs GLA-AOT vs. Shea SLNs GLA-TRITC-AOT; ççç Shea SLNs GLA-TRITC-AOT vs. Mango SLNs GLA-TRITC-AOT. Two-tailed unpaired *t*-test: left vs. right panel (just prepared vs. 30 days): *** Mango SLNs GLA-TRITC-AOT; * Mango SLNs GLA-AOT. Figure S5: GLA recovery in freeze-dried powders and tablets (from Table 3 and Table 4). Statistical analysis: one-way ANOVA, followed by Bonferroni’s multiple comparison test. Left panel: *** suspension vs. 1:1; * suspension vs. 2:1; ° 1:1 vs. 2:1; °° 1:1 vs. 4:1. Two-tailed unpaired *t*-test: right panel: * Shea suspension vs. Mango suspension. Figure S6: Mean size of freeze dried powders and tablets (from Table 3 and Table 4). Statistical analysis: one-way ANOVA, followed by Bonferroni’s multiple comparison test: right panel: Shea series: ** Suspension vs. tablet formulations. Two-tailed unpaired *t*-test: right panel: °°° Shea suspension vs. Mango suspension. Figure S7: Redispersion and disaggregation times of freeze-dried powders and tablets (from Table 3 and Table 4). Statistical analysis: one-way ANOVA, followed by Bonferroni’s multiple comparison test: upper left panel: *** 1:1 vs. all other formulations. Two-tailed unpaired *t*-test: upper right panel: Shea series: * Plain Tablet vs. Tablet + Eff.; Mango series: ° Plain Tablet vs. Tablet + Eff.
