# Supplementary material for: Solid Lipid Nanoparticles by Coacervation from Natural Soaps: Preliminary Studies for Oral Delivery of an Insulin Analogue
Source: Pharmaceutics. 2025 Sep 26;17(10):1261. doi: 10.3390/pharmaceutics17101261 (PMC12567550; doi:10.3390/pharmaceutics17101261)
Supplement: Supplementary file 1 [file pharmaceutics-17-01261-s001.zip › pharmaceutics-3853546-supplementary.pdf]

Supplementary Materials

Solid Lipid Nanoparticles by Coacervation from Natural Soaps: Preliminary Studies for Oral Delivery of an Insulin Analogue

Annalisa Bozza, Arianna Marengo, Federica Blua, Elisabetta Marini, Stefano Bagatella, Elena Ugazio, Elisabetta Muntoni, and Luigi Battaglia

Abbreviation list **page 2**

Figure S1 **page 3**

Figure S2 **page 4**

Figure S3 **page 5**

Figure S4 **page 6**

Figure S5 **page 7**

Figure S6 **page 8**

Figure S7 **page 9**

Abbreviation list: AOT: sodium docusate; EE%: % entrapment efficiency; Eff.: effervescent mixture; GLA: glargine insulin; SLNs: solid lipid nanoparticles; TRITC: tetramethylrhodamine isothiocyanate

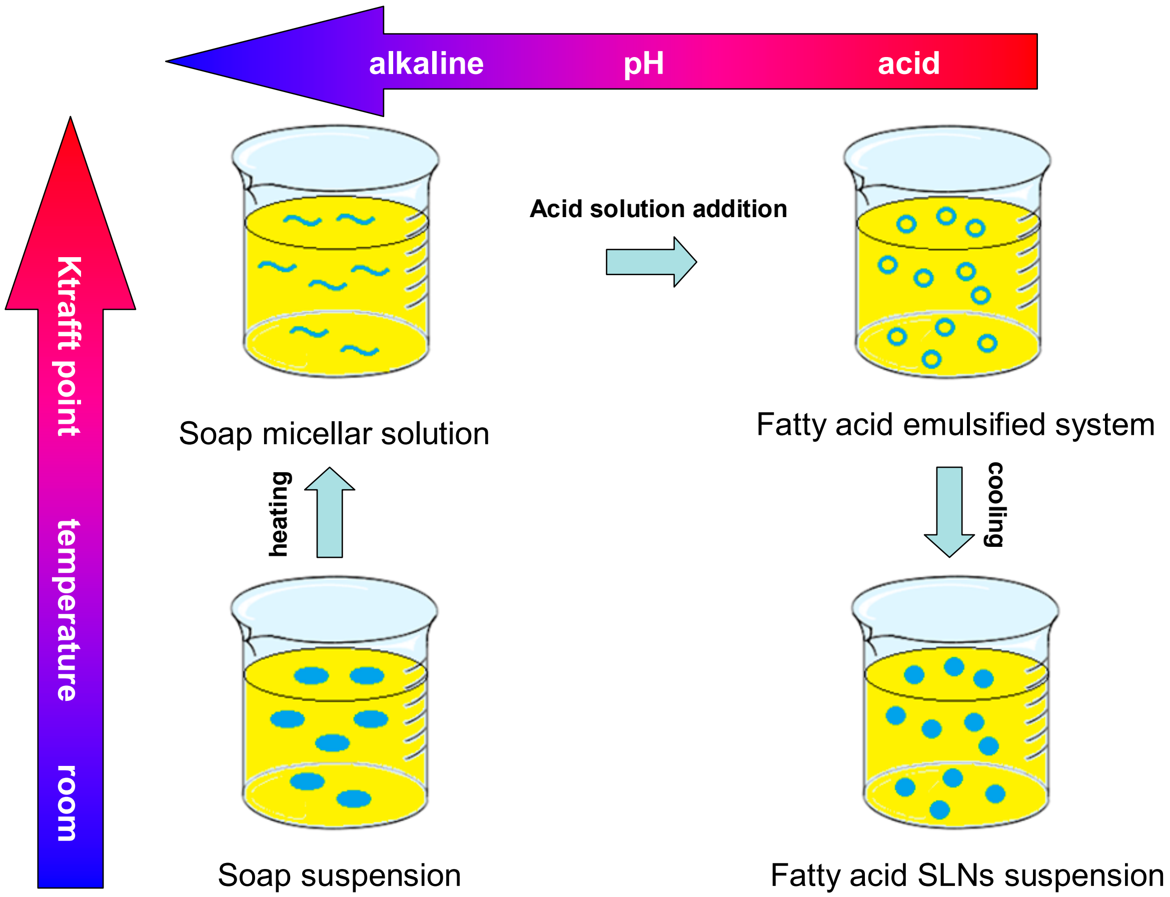

Figure S1: scheme of the fatty acid coacevation.

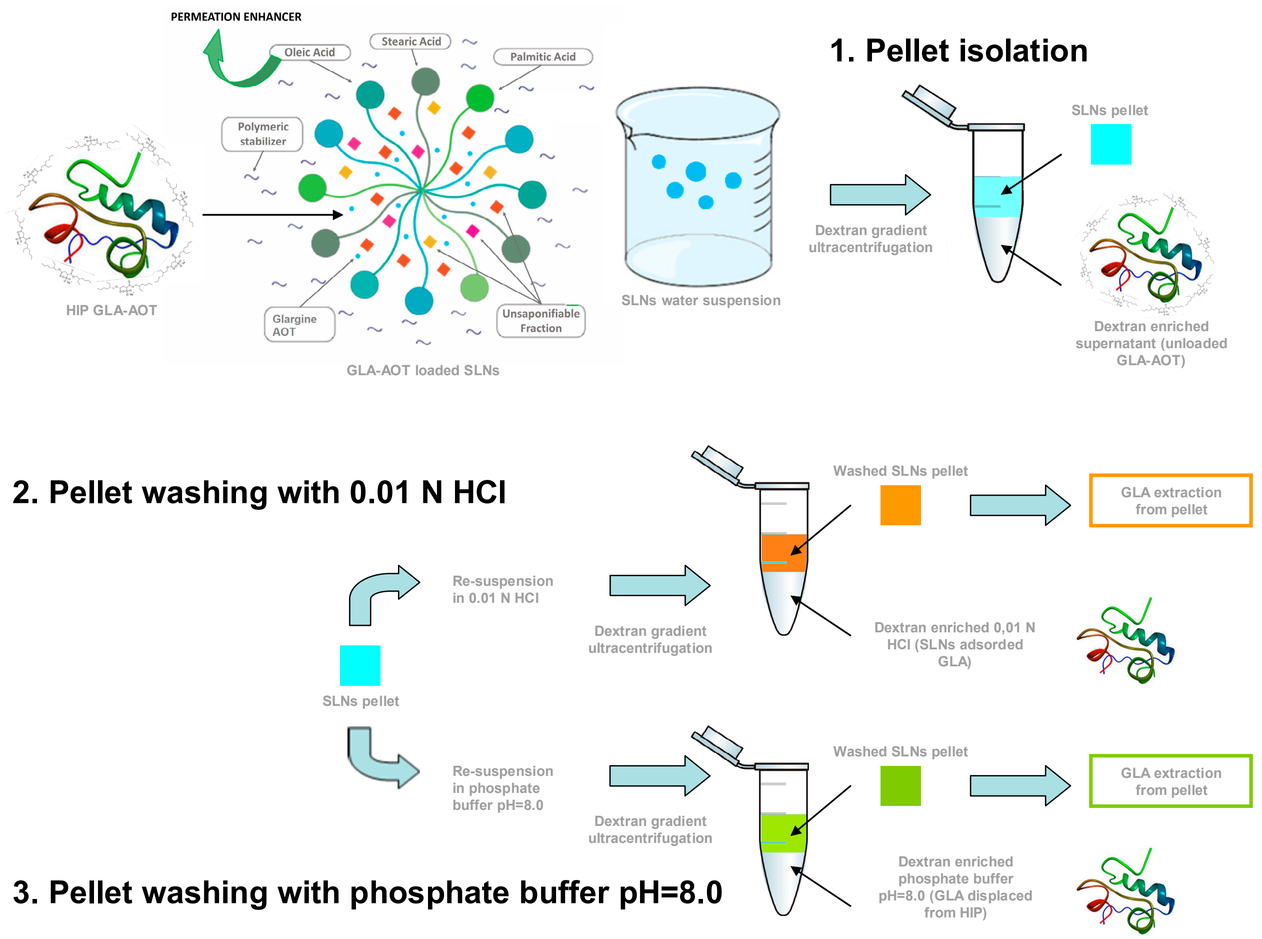

Figure S2: Scheme of the extraction procedure of GLA from SLNs

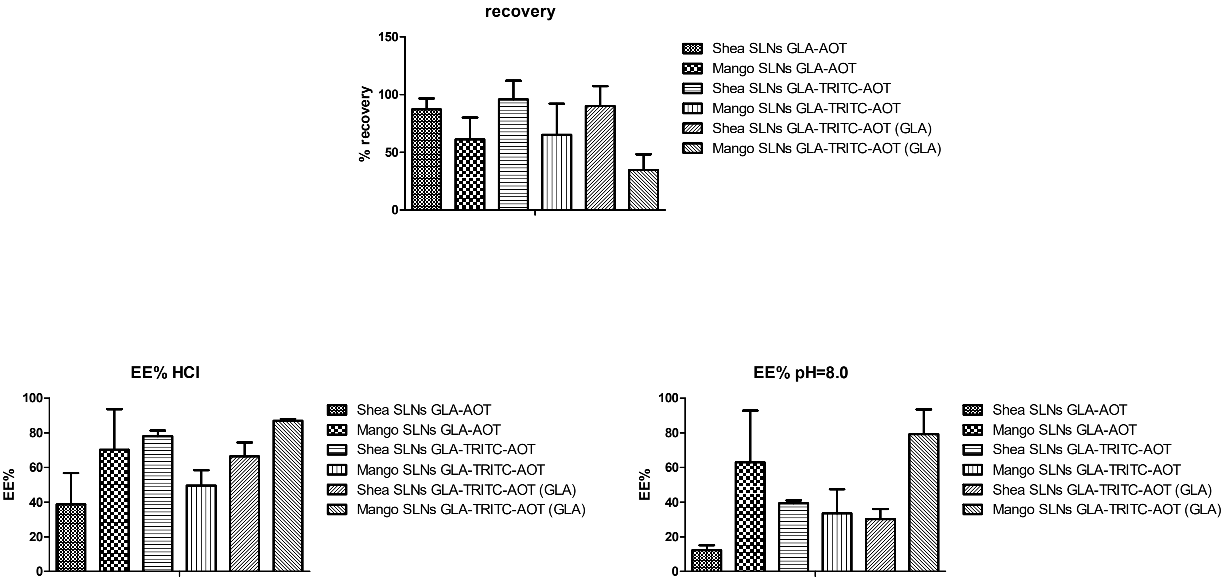

Figure S3: Recovery and EE% of GLA-AOT and GLA-TRITC-AOT loaded SLNs (from Table 2).

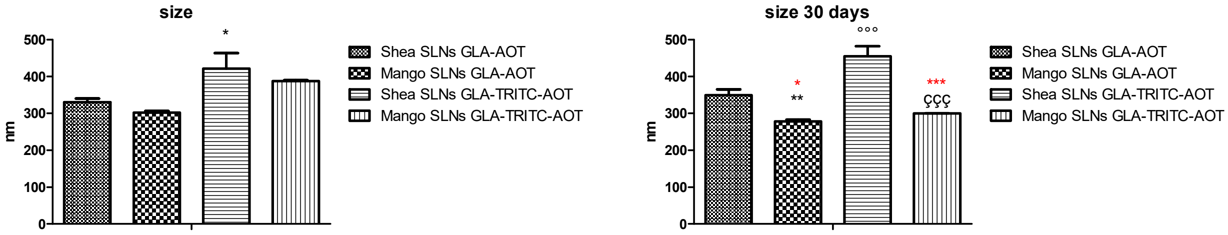

Figure S4: Mean size of GLA-AOT and GLA-TRITC-AOT loaded SLNs (from Table 2). Statistical analysis: one-way ANOVA, followed by Bonferroni's multiple comparison test. Left panel: * Mango SLNs GLA-AOT vs Shea SLNs GLA-TRITC-AOT; right panel: ** Shea SLNs GLA-AOT vs Shea SLNs GLA-TRITC-AOT; °°° Mango SLNs GLA-AOT vs Shea SLNs GLA-TRITC-AOT; ççç Shea SLNs GLA-TRITC-AOT vs Mango SLNs GLA-TRITC-AOT. Two tailed unpaired T-test: left vs right panel (just prepared vs 30 days): *** Mango SLNs GLA-TRITC-AOT; * Mango SLNs GLA-AOT.

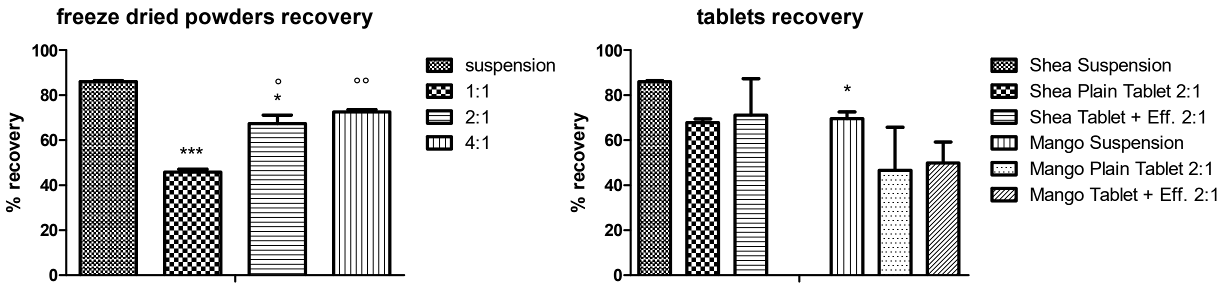

Figure s5: GLA recovery in freeze dried powders and tablets (from Tables 3 and 4). Statistical analysis: one-way ANOVA, followed by Bonferroni's multiple comparison test. Left panel: *** suspension vs 1:1; * suspension vs 2:1; ° 1:1 vs 2:1; °° 1:1 vs 4:1. Two tailed unpaired T-test: right panel: * Shea suspension vs Mango suspension.

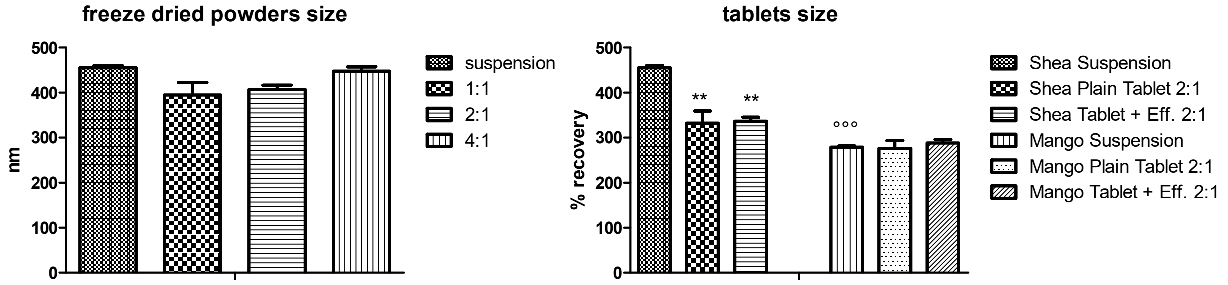

Figure S6: Mean size of freeze dried powders and tablets (from Tables 3 and 4). Statistical analysis: one-way ANOVA, followed by Bonferroni's multiple comparison test: right panel: Shea series: ** Suspension vs tablet formulations. Two tailed unpaired T-test: right panel: °°° Shea suspension vs Mango suspension.

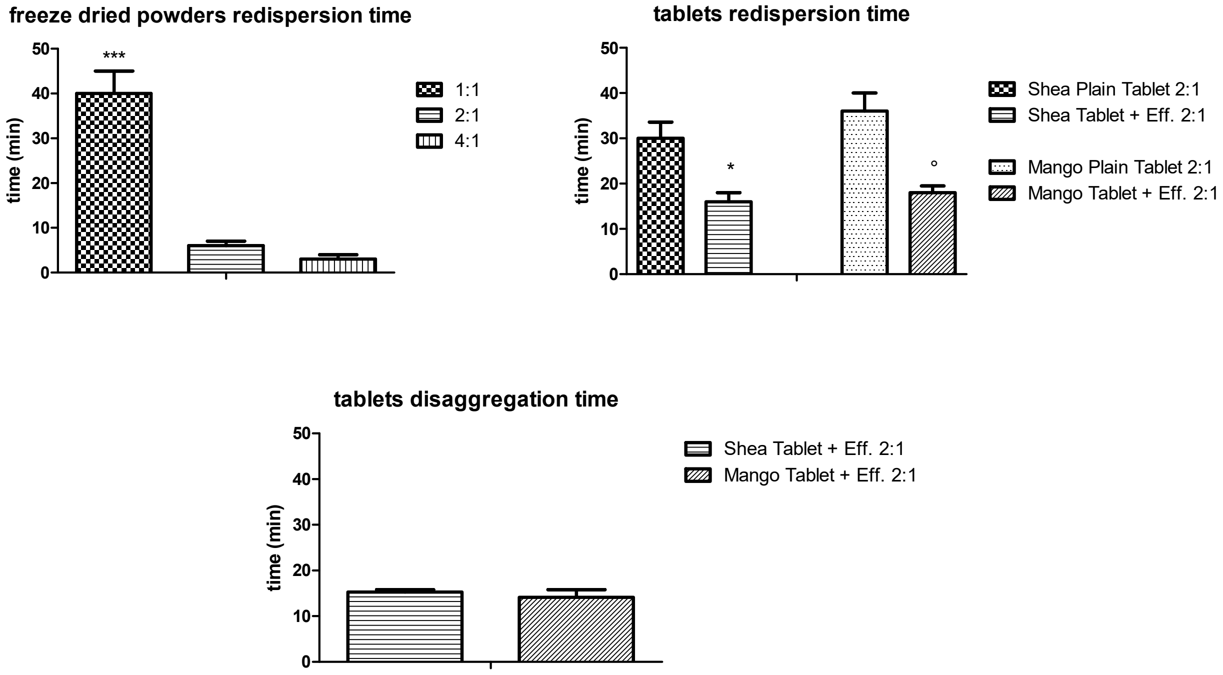

Figure S7: Redispersion and disaggregation times of freeze dried powders and tablets (from Tables 3 and 4). Statistical analysis: one-way ANOVA, followed by Bonferroni's multiple comparison test: upper left panel: *** 1:1 vs all other formulations. Two tailed unpaired T-test: upper right panel: Shea series: * Plain Tablet vs Tablet + Eff.; Mango series: ° Plain Tablet vs Tablet + Eff.
